# Tremor Detection Using Parametric and Non-Parametric Spectral Estimation Methods: A Comparison with Clinical Assessment

**DOI:** 10.1371/journal.pone.0156822

**Published:** 2016-06-03

**Authors:** Octavio Martinez Manzanera, Jan Willem Elting, Johannes H. van der Hoeven, Natasha M. Maurits

**Affiliations:** Department of Neurology, University Medical Center Groningen (UMCG), University of Groningen, Groningen, the Netherlands; Duke University, UNITED STATES

## Abstract

In the clinic, tremor is diagnosed during a time-limited process in which patients are observed and the characteristics of tremor are visually assessed. For some tremor disorders, a more detailed analysis of these characteristics is needed. Accelerometry and electromyography can be used to obtain a better insight into tremor. Typically, routine clinical assessment of accelerometry and electromyography data involves visual inspection by clinicians and occasionally computational analysis to obtain objective characteristics of tremor. However, for some tremor disorders these characteristics may be different during daily activity. This variability in presentation between the clinic and daily life makes a differential diagnosis more difficult. A long-term recording of tremor by accelerometry and/or electromyography in the home environment could help to give a better insight into the tremor disorder. However, an evaluation of such recordings using routine clinical standards would take too much time. We evaluated a range of techniques that automatically detect tremor segments in accelerometer data, as accelerometer data is more easily obtained in the home environment than electromyography data. Time can be saved if clinicians only have to evaluate the tremor characteristics of segments that have been automatically detected in longer daily activity recordings. We tested four non-parametric methods and five parametric methods on clinical accelerometer data from 14 patients with different tremor disorders. The consensus between two clinicians regarding the presence or absence of tremor on 3943 segments of accelerometer data was employed as reference. The nine methods were tested against this reference to identify their optimal parameters. Non-parametric methods generally performed better than parametric methods on our dataset when optimal parameters were used. However, one parametric method, employing the high frequency content of the tremor bandwidth under consideration (High Freq) performed similarly to non-parametric methods, but had the highest recall values, suggesting that this method could be employed for automatic tremor detection.

## Introduction

Tremor is defined as a rhythmical, involuntary oscillatory movement of a body part [1]. Its diagnosis is mainly a clinical process where patients are interviewed, and undergo clinical observation and testing. Tremor of the affected body part(s) may be further evaluated during diagnostic assessment which typically includes quantification of tremor characteristics using accelerometers (ACC), and/or electromyography (EMG). Such further assessment of tremor involves a procedure that lasts approximately 30 minutes in which the patient is instructed to perform certain tasks by a clinician or technician while ACC and/or EMG data are recorded from the affected body part(s). Subsequently, the recorded ACC and EMG signals are analyzed offline by a clinician to obtain quantitative parameters that characterize tremor, such as frequency (variability), intermittency, extent and laterality. One of the most important parameters is the dominant tremor frequency. This parameter has been the focus of many attempts to discern tremor etiology [2]. Frequency analysis can be used to determine the dominant tremor frequency, by calculating the power spectral density (PSD) of the signal. This technique has previously been applied to tremor data to differentiate between different disorders, with good results [2,3].

Tremor is the most common movement disorder symptom, and includes tremor in Parkinson’s disease (PD), essential tremor (ET), enhanced physiological tremor (EPT), dystonic tremor (DT), orthostatic tremor (OT) and functional tremor (FT). The latter is the most common functional movement disorder [4,5]. Each of these forms of tremor has typical characteristics. For example, PD tremor is typically a rest tremor with a relatively stable dominant frequency of 4–8 Hz [6]. While FT has a similar frequency range to PD tremor, FT typically has greater frequency variability. Furthermore, FT can be recognized by its typical amplitude increase when the patient pays attention to the symptom and amplitude reduction and even tremor cease when the patient is distracted from the symptom. For FT in particular, these variations in tremor presentation and the time limitations of the diagnostic assessment hinder its diagnosis. Longer observations of tremor may render the diagnosis of FT more reliable. Self-reported tremor has been used to monitor tremor outside the clinic, however, for FT there is a characteristic mismatch between these reports and objective assessments of tremor [7]. Long-term recordings [8] in the home environment can be helpful to obtain an objective tremor assessment and to detect variations in the presentation of tremor that cannot be observed in the clinic. However, an inconvenience of long-term recordings is the large amount of data generated. These data cannot be easily assessed using the same visual inspection methodology that is applied to more typical short term recordings as it would take too much time.

Therefore, we investigated techniques that automatically detect tremor segments in ACC data, as this type of data is more easily obtained in the home environment than EMG data. Time can be saved if clinicians only evaluate the tremor characteristics of segments that have been automatically detected in longer daily activity recordings.

In the present study, we therefore evaluated automatic tremor detection methods that are suited for evaluating long-term ACC recordings. To detect segments containing tremor, the signal was divided into segments of short length. Each segment was classified individually as “tremor” or “no tremor” according to its PSD estimate and its dominant frequency. There are many techniques to estimate the PSD of a signal. In this study we compared the performance of classical (also called non-parametric) and parametric methods to estimate the PSD of the signal, which constitutes the primary component of the detection algorithm. Classical methods employ the Fast Fourier Transform (FFT). Classical methods have been widely used in clinical trials [9] even though it is known that these methods produce PSDs with poor frequency resolution when applied to short segment lengths. An alternative is provided by parametric methods, which characterize a time series signal by a model. In contrast to classical methods, parametric methods do not have limited frequency resolution. Parametric methods assume that the time series signal can be described by a model plus white noise. One of the parameters that has to be defined is the order of the model that characterizes the signal. Salarian et al. [8] studied the automatic detection of tremor using parametric methods on signals acquired from gyroscopes. In their study the PSD was estimated using a 6^th^ order autoregressive (AR) model with good results, but a justification for this order selection was not provided. Moreover, the gold standard for tremor scoring used by Salarian et al. employed video recordings and they found that video recordings do not always provide optimal images for classifying tremor (especially at low amplitudes), resulting in possible misclassification [8]. A comparison between classical and parametric methods for the analysis of tremor data was presented by Spyers-Ashby et al. (1998). However, they only analyzed two tremor recordings of short duration. We employed the consensus on tremor assessment between two clinicians to define the reference in the present study. Clinical assessment of tremor was performed similarly to the normal clinical procedure, but as the goal is to develop an algorithm that can be used on long term recordings from the home environment, only ACC and not EMG signals were used in the clinical assessment. We did not choose an a priori model order for parametric methods, but evaluated different model order selection criteria instead. A method for automatic tremor detection should not depend on the specific tremor investigated. Therefore, we evaluated the methods on a population of patients with different forms of tremor present in the hands as a minimum requirement.

To our knowledge, the present study is the first to compare the results of different automatic methods with the visual assessment of tremor in ACC data. We aimed to investigate whether an automatic method can be used to detect tremor segments in long-term and short-term recordings with similar performance to the evaluation of experienced clinicians (as expressed in a maximal F_1_ score [10]) using only ACC data. Such a method would provide an important step towards the evaluation of home environment tremor monitoring using accelerometers, saving valuable time by allowing a clinical assessor to focus only on segments containing tremor.

## Materials and Methods

### Participants

We retrospectively analyzed ACC recordings obtained between December 2008 and January 2013 from the diagnostic assessments of 14 patients with different forms of (at least) hand tremor at the University Medical Center in Groningen (Table 1). We did not specify the disorder a priori, but rather tried to include as many different forms of tremor as possible as we focused on detecting any type of tremor. Patient records were anonymized and de-identified prior to analysis.

**Table 1:** Patient characteristics.

Patient	Diagnosis	Sex	Age (years)	Recording (minutes)
1	Functional tremor	Male	67	23.3
2	Essential tremor	Male	81	16
3	Enhanced Physiological tremor	Male	47	28.9
4	Enhanced Physiological tremor	Male	59	19.5
5	Essential tremor	Female	75	18.2
6	Essential tremor	Male	66	22
7	Essential tremor	Male	76	15.2
8	Parkinsonian tremor	Female	75	15.7
9	Ataxia	Female	62	32
10	Parkinsonian tremor	Male	75	32.5
11	Functional tremor	Male	60	27
12	Parkinsonian tremor	Male	59	30
13	Parkinsonian tremor	Male	82	15.5
14	Parkinsonian tremor	Male	65	14

Diagnosis, sex, age and tremor recording duration of each patient.

### Data Acquisition

The signal used for analysis was obtained from routine clinical assessments employing a uniaxial accelerometer placed on the dorsal side of the hand of the most affected limb. The data were sampled at a frequency of 1 kHz (mean duration = 23.1 minutes, Table 1) while patients performed a number of tasks that may evoke tremor (for example: rest, hand and arm extension to the front and to the side, finger chasing, finger-to-nose movements, diadochokinesis) in different positions and while occasionally being distracted from the task. To evaluate the automatic detection methods and to facilitate tremor scoring by the clinicians, relatively short term recordings were selected, but the methods presented here can be applied to long term recordings without modifications. The data were analyzed offline with Matlab (version 7.12, Mathworks, Natick, Massachusetts, U.S.A). A high pass filter (FIR, 4^th^ order Butterworth, cutoff frequency = 0.25 Hz) and a low pass filter (FIR, 2^nd^ order Butterworth, cutoff frequency = 45 Hz) were applied before segmentation and PSD estimation to suppress movement artifacts and mains artifact, respectively.

### Clinical Tremor Classification

**Fig 1:**
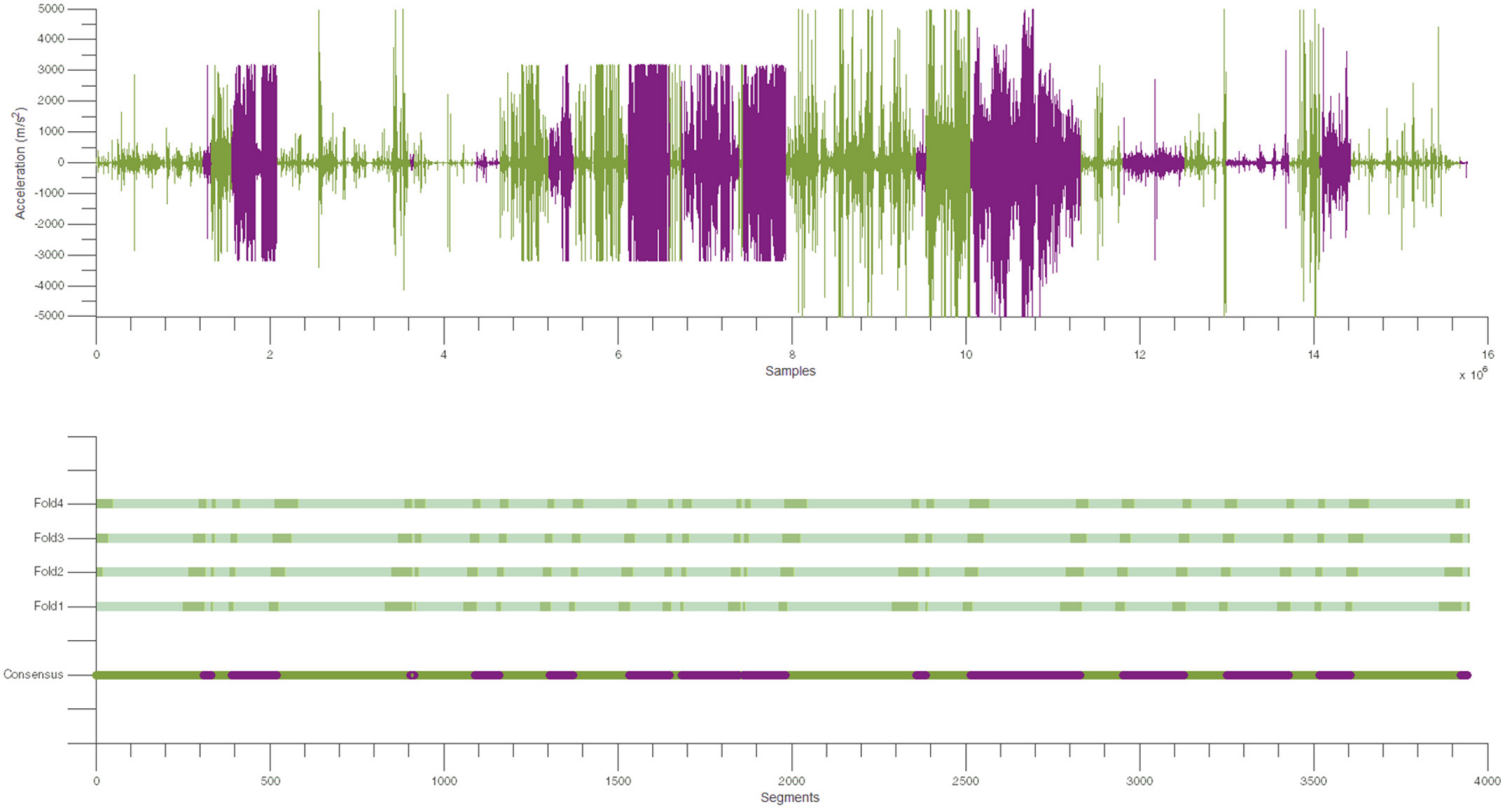
Building the consensus signal. Top: The signal to be analyzed is constructed from segments where two clinicians agreed on the presence or absence of tremor. These segments were concatenated. This signal is composed of the tremor segments (in purple) and the “no tremor” segments (in green) of the 14 patients. It is important to note that the segments were first rearranged for each patient, in the following order: all “no tremor” segments followed by all “tremor” segments. Bottom: The bottom signal is the consensus between both clinicians. Above this signal, the four iterations used to evaluate each method are described. For each iteration, dark green segments represent the segments employed to obtain the optimal parameters and light green segments represent the segments employed to evaluate the optimal parameters.

The accelerometer signal was divided into segments of four seconds, for visual identification by clinicians. Two clinical neurophysiologists (JWE and JHvdH) with ample experience in tremor assessment, independently classified each individual segment as “tremor” or “no tremor” depending on qualitative and quantitative aspects of the signal. Initially, each segment was visually assessed. If considered necessary, a PSD tool (based on FFT) was applied to the segment to evaluate the spectral content. This approach was chosen to replicate the evaluation performed during routine assessments of tremor recordings. During routine assessment ACC and EMG signals are simultaneously assessed. In this study, only ACC signals were considered because ACC recordings are much more suitable than EMG recordings for monitoring tremor in the home environment. The segment classifications of both clinicians were employed to define the gold standard reference. This reference was composed of segments that were given the same classification by both clinicians. Consequently, only the signal segments for which there was consensus on the presence or absence of tremor were analyzed (3943 segments, corresponding to 81% of all data). The rest of the segments were discarded. Median tremor prevalence per patient was 46.12% (range 2.53–89.7%). The goal of this study was to compare the performance of different tremor detection methods and to identify their optimal parameters, independent of the type of tremor. Therefore, the segments of all patients for which there was consensus were concatenated, forming a single long signal (Fig 1). This made it possible to evaluate tremor detection performance regardless of the disorder of the patient.

### Automatic Tremor Classification

Tremor detection methods classified each segment as “tremor” or “no tremor” based on two inter-related parameters derived from the PSD of each segment: the dominant frequency (DF) of the PSD estimation and the amplitude of the DF. For most methods, the DF was defined as the frequency with highest amplitude in the PSD.

### PSD Estimation

Both parametric and classical methods can be used to estimate the frequency content of a signal [3]. Parametric methods have the advantage of no frequency resolution limitations when applied to segments of short length; instead the structure of the signal is characterized by a model. We selected the autoregression method using Burg’s approach [11] to represent parametric methods and the modified periodogram and the Welch method [12] to represent classical methods. The reasons for choosing these methods, their advantages and their limitations are explained below.

#### Parametric PSD estimation methods

Parametric methods represent the time series signal by a model plus white noise. Before explaining the Burg method we first summarize general autoregressive (AR) models. AR models can model signals that are well characterized by the peaks in the signal spectrum. Since this is a characteristic of tremor data which is locally highly periodic, AR methods can be thought of as the most appropriate methods to analyze tremor data [3]. AR modeling of a time series is based on the assumption that each value of the series can be predicted as a weighted sum of previous values of the same series plus an error term [13]:

$$x[n]=\sum_{i=1}^{k}a_{i}x[n-i]+\varepsilon [n]$$ (1)

where *x[n]* is the current value of the time series, *a*_i_ is the predictor coefficient i, *ε* is the prediction error and *k* is the order of the model.

There are different methods to calculate the predictor coefficients *a*_*i*_. The most commonly used are the covariance method and the Yule-Walker method. The predictor coefficients form the model filter. By transforming the model using the z-transform an interpretation in terms of time delay is obtained: after z-transformation the effect of a time shift by *i* sampling-interval-time units is expressed as multiplication by *z*^−*i*^ [14]. After z-transformation the model can thus be expressed as:

$$X[z]=\sum_{i=1}^{k}a_{i}X[z]z^{-i}+W[z]$$ (2)

which is equivalent to:

$$\frac{X(z)}{W(z)}=\frac{1}{1-\sum_{i=1}^{k}a_{i}z^{-i}}=H(z)$$ (3)

where H(z) is the AR synthesis filter. A synthesis filter transforms the flat spectrum of the (white) noise input W(z) into a shape similar to the one of the original spectrum [15]. X(z) can be regarded as the output of the AR-filter applied to the prediction error sequence W(z) [13]. From the frequency response of the AR-filter we can estimate the DF of the original time series signal by identifying the frequency that has the highest peak in the PSD.

#### Burg method

The Burg method is an AR method in which each value of a series is predicted on the basis of both its past values (forward prediction) and its future values (backward prediction), *y[n]* and *z[n]* in Eq (4). The Burg method guarantees the stability of the synthesis filter and outperforms the Yule-Walker and covariance methods on short data records [16]. To obtain the coefficients of the filter, Burg’s minimization criterion minimizes the sum of the squares of both the forward and the backward squared prediction errors [16], *F*_*k*_ and *B*_*k*_ in Eq (5) [17].

$$y[n]=-\sum_{i=1}^{k}a_{i}x[n-i],\;z[n]=-\sum_{i=1}^{k}a_{i}x[n+i]$$ (4)

$$F_{k}={\sum_{n=k}^{N}\left(x_{n}-y_{n}\right)}^{2},\;B_{k}={\sum_{n=k}^{N}\left(x_{n}-z_{n}\right)}^{2}$$ (5)

In Eq (4), *y[n]* is the forward linear prediction for sample *n*, *z[n]* is the backward linear prediction for sample *n*, and *k* is the order of the model. In Eq (5), *x[n]* is the sample *n*, *F*_*k*_ is the forward linear prediction error, *B*_*k*_ is the backward linear prediction error, and *N* is the length of the time series.

#### Model order

The number of values used in the prediction is called the model order [13] and is equivalent to the number of coefficients that are used to describe the model. The selection of the optimal order is not straightforward. If the order is too low then the estimated spectra might not have enough detail (peaks might be missing), and if the order is too high a single peak in the PSD estimation might bifurcate. A wide range of order selection criteria can be found in literature but there is no guarantee that any selection criterion will perform well under all circumstances [16]. With this argument in mind we decided to evaluate the frequency response of each AR model from the 1^st^ to the 20^th^ order. We considered that 20 models were sufficient for the evaluation of the frequency response because this number provided a good compromise between the number of models evaluated and the calculation time. Subsequently, we estimated the DF with five different selection criteria, as there is not a superior criterion defined in the literature.

#### Dominant frequency estimation

**Fig 2:**
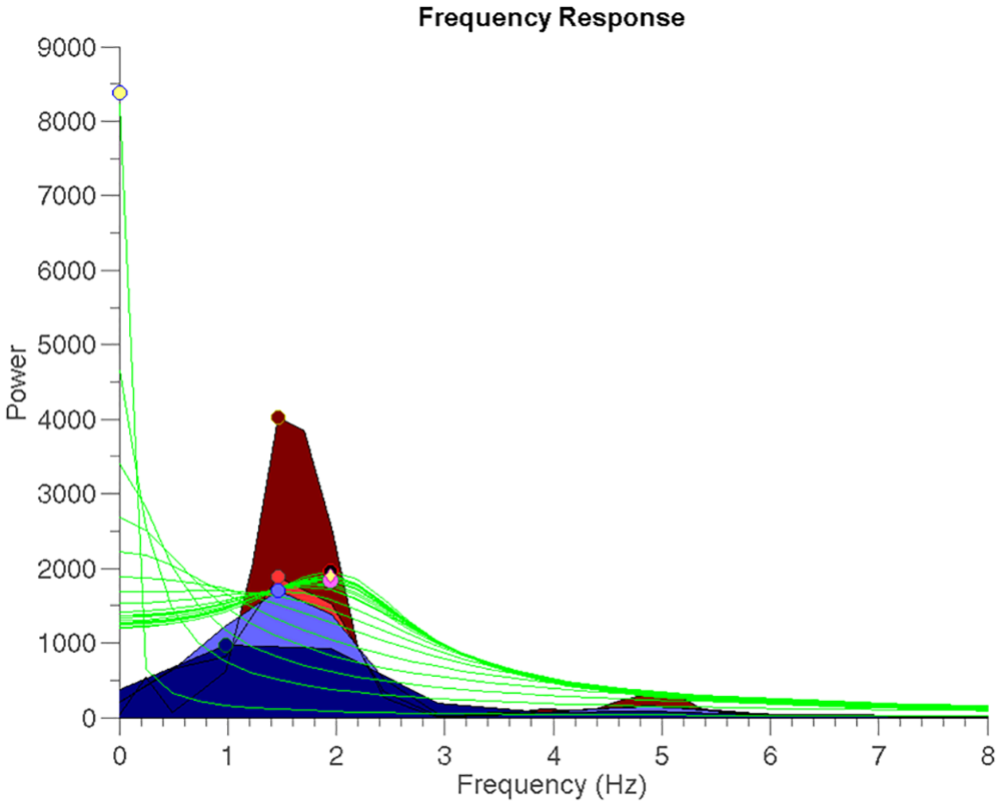
Frequency responses of autoregressive models. Frequency responses of 20 AR models (1^st^ to 20^th^ order) of one segment of 4 s (green lines). The DFs were determined according to five criteria: 1) *High Amp*: the DF corresponds to the frequency of the highest peak among all frequency responses (yellow dot). 2) *High Freq*: the DF corresponds to the frequency of the peak with the highest frequency among all frequency responses (pink dot). 3) *Akaike*: the DF corresponds to the frequency of the highest peak of the model selected by Akaike criterion (black dot). 4) *Mode High Amp*: the DF corresponds to the value of the mode of the frequencies of the highest peak of each model (yellow diamond). 5) *Mode High Freq*: the DF corresponds to the value of the mode of the frequencies of the peak with the highest frequency of each model (yellow diamond). Note that for this segment Mode High Amp and Mode High Freq have the same value. The PSD employing the periodogram, Welch (2), Welch (3) and Welch (8) methods are shown in dark red, red, blue and dark blue respectively. Also, the DF of each method is shown with a dot of its corresponding color.

Voluntary human movements and involuntary tremor both produce movements of relatively low frequency. Therefore we limited PSD estimation to the frequency range from 0 to 20 Hz. We explored five criteria to determine the DF of each segment within this range (Fig 2). For all criteria the frequency responses of all models (1^st^ to 20^th^ order) were analyzed. The DF was selected according to the following five criteria.

1. ***Akaike***: Only one model is selected from all possible AR models [18]. This criterion is a measure of how well a model fits the original data. It also penalizes for a large number of parameters to avoid high model complexity. The DF was determined as the frequency that corresponds to the peak with the highest amplitude of the frequency response in the 0 to 20 Hz band (frequency related to black dot in Fig 2).
2. ***High Amp***: The DF was determined as the frequency that corresponds to the highest amplitude in the spectra from all 20 models (frequency corresponding to yellow dot in Fig 2).
3. ***High Freq***: The DF was determined as the frequency that corresponds to the peak with the highest frequency (within the DC-20 Hz band) in the spectra from all 20 models (frequency corresponding to pink dot in Fig 2).
4. ***Mode High Amp*:** The DF was determined as the mode of the frequencies that correspond to the highest amplitude in each of the spectra from all 20 models (frequency corresponding to yellow diamond in Fig 2).
5. ***Mode High Freq***: The DF was determined as the mode of the frequencies that correspond to the peak with the highest frequency in each of the spectra from all 20 models (frequency corresponding to yellow diamond in Fig 2).

Two of these methods determine the DF as the mode of the DFs of each frequency response according to a specific criterion. Most models will accurately represent the frequency response of the original signal, only a few will not. The mode was chosen to ensure that we discarded the models that were not representative of the data. Very high order models might not be realistic for tremor data, but their inclusion in the analysis improved the methods employing the mode.

#### Classical PSD estimation methods

There are many methods that estimate the PSD using FFT. The modified periodogram is one of the less computationally expensive methods. However, it suffers from various disadvantages, such as high variance. The Welch method is an adaptation of the modified periodogram. By taking the PSD of overlapping windows and averaging their values the variance of the PSD is reduced [3]. In this study, we treated each data segment as a set of data independent from the rest of the tremor recording. By splitting these segments into smaller (overlapping) windows a series of spectral estimates was obtained. The average of those estimates can be employed to reduce the variance [3]. Increasing the number of windows over which the average is taken decreases the error variance, but also reduces the spectral resolution. In order to reduce the variance while maintaining an acceptable PSD resolution, we studied the Welch method using two, three and eight windows with a 50% overlap for each segment (further referred to as Welch (2), Welch (3) and Welch (8) methods).

### Tremor Bandwidth and Power Threshold

Each segment was classified as “tremor” or “no tremor” according to the DF characteristics of the segment. For the most prevalent tremors it is known that the DF varies between 4–12 Hz [19]. Patients with a tremor-dominant form of PD and patients with ET may present with a tremor frequency of 4–8 Hz [6]. Patients with dystonic tremor usually show focal tremor with irregular amplitude and variable frequency (typically less than 7 Hz) [1]. However, tremor can only be identified if the amplitude of the DF exceeds a certain value. Therefore, a segment was classified as “tremor” only if its DF was within a specific bandwidth and if the amplitude of the DF in the PSD estimate was larger than a specific threshold. For tremor bandwidth, the upper limit was set to 12 Hz and the lower limit was varied from 0 to 12 Hz in steps of 0.05 Hz. The power threshold was varied from 0 to 5000 m^2^/s^4^ in steps of 50 m^2^/s^4^.

### Optimal Parameters and Cross-Validation

For diagnostic purposes, clinicians are more interested in “tremor” segments than in “no tremor” segments. Therefore, we chose metrics that focus on the accurate detection of “tremor” segments to evaluate the performance of the automatic tremor detection methods. Two metrics that can be employed are precision and recall. For this study, precision relates to the number of segments classified as “tremor” that truly are tremor compared to the total number of segments that were classified as “tremor”, while recall relates to the number of segments classified as “tremor” that truly are tremor compared to the total number of segments that truly are tremor. Depending on the type of tremor being analyzed one metric could be more important than the other. However, for this initial approach and since there is no evidence to favor one metric we decided to treat both metrics equally [20]. The F_1_ score, which combines and gives equal weight to precision and recall, has been used previously to evaluate tremor detection performance [21] and can be employed to identify the optimal parameters. The F_1_ score allows the identification of the optimal algorithm parameters using a single number [10] and is given by:

$$F_{1}=2*\frac{precision*recall}{precision+recall}$$ (6)

Receiver operating curves (ROC) and precision-recall (PR) curves were employed to evaluate the performance of each method. It is important to note that these different approaches might result in different optimal parameters [22]. The parameters that correspond to the point closest to the (0, 1) coordinate of the ROC curve were also obtained for comparison.

To obtain an estimate of the performance of each method on new data, four-fold cross validation was employed. For each iteration, a subset containing 80% of all segments (80% of the “tremor” segments and 80% of the “no tremor” segments) was employed to determine the optimal parameters according to the F_1_ score. The optimal lower limit of the tremor bandwidth and power threshold values of each method were identified by averaging the F_1_ scores for all possible combinations of parameters for each iteration. After their identification, each method was tested using the optimal parameters, on the remaining 20% of the data (20% of the “tremor” segments and 20% of the “no tremor” segments). The mean performance of each method on the test data provided an estimate of the performance of each method on new data. To obtain an unbiased estimate, we verified that the subsets corresponding to 80% of the data used to obtain the optimal parameters always included 80% of the “tremor” segments and 80% of the “no tremor” segments for each patient and similarly for the 20% test subsets. This process is illustrated in Fig 1.

## Results

**Fig 3:**
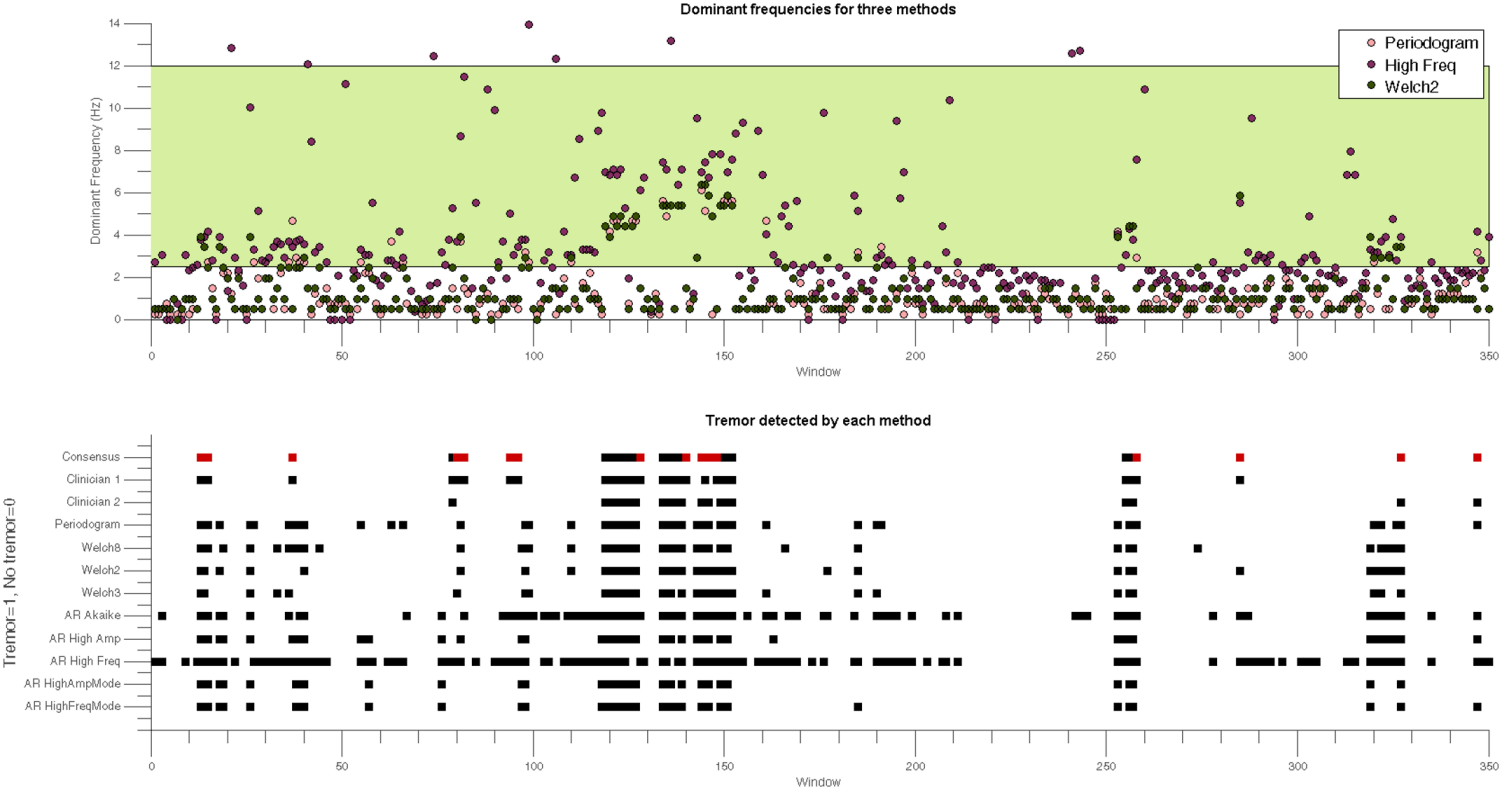
Example of dominant frequencies and tremor classification of each segment for patient 1. Top: identified DFs during the analysis of approximately 23 minutes of tremor data using *Periodogram* (pink), *High Freq* (purple) and Welch (2) (green). Each circle corresponds to the dominant frequency of one segment. This figure illustrates that the High Freq method was not constrained to a specific frequency resolution like the Periodogram or Welch methods. A segment was classified as “tremor” if the DF was located in the 2.5–12 Hz frequency band (pale green) for this example. Bottom: The tremor classification according to the consensus between both clinicians (black: agreement on tremor presence, white: agreement on tremor absence, red: lack of agreement), and for every automatic detection method are shown for the same dataset (tremor bandwidth = 2.5–12 Hz, power threshold = 0 m^2^/s^4^). The tremor classification corresponds to individual segments, before rearranging the data (as illustrated in Fig 1).

To illustrate our approach, we have plotted results for three methods for Patient 1 in Fig 3 (top). For this patient, segments that the clinicians agreed on accounted for 95% of all segments. The average agreement across all subjects was 81%. Fig 3 shows that, in contrast with classical methods, parametric methods are not constrained to a limited number of frequencies. For every patient, we also plotted the agreement between the two clinicians together with the results from the automatic tremor detection methods for visual inspection (as illustrated for Patient 1 in Fig 3, bottom).

### Methods Performance

**Fig 4:**
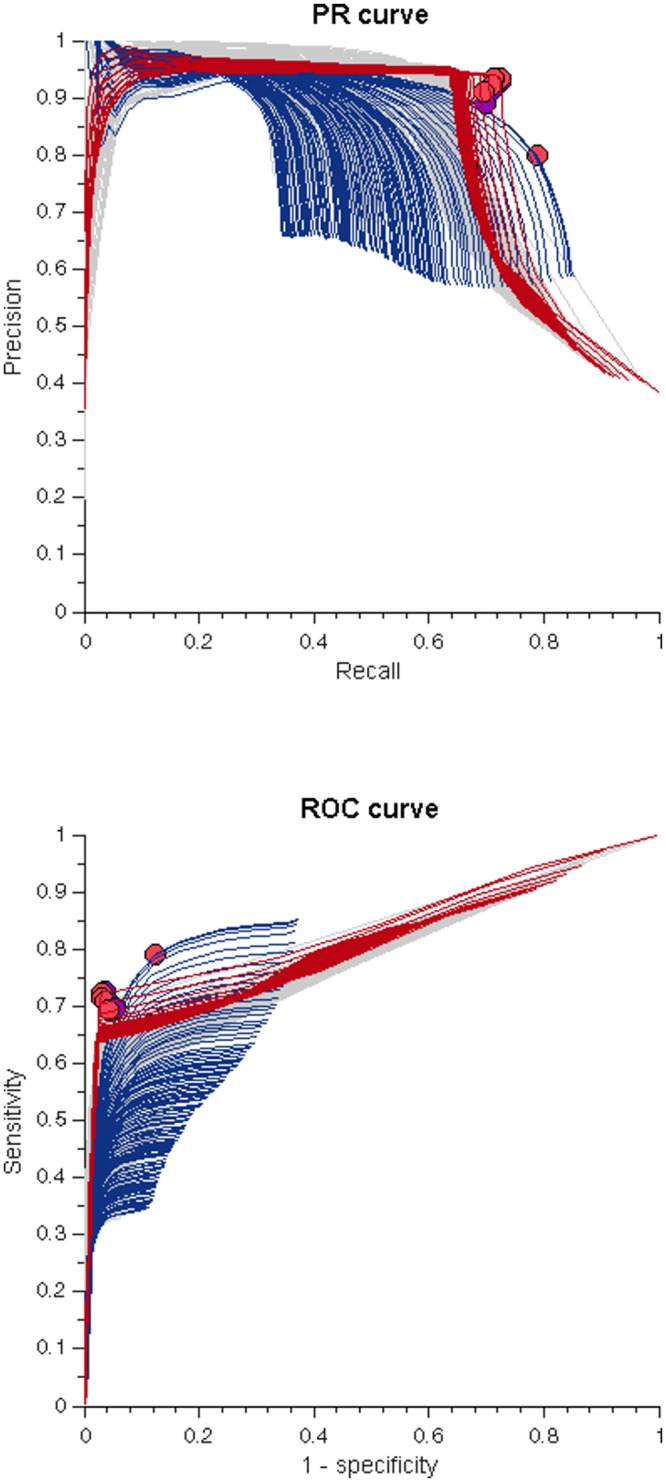
ROC and PR curves for each method for one of the training folds. Top: Precision and Recall are employed to build the PR curves of each method (gray lines). The optimal point of each method according to these metrics is plotted with a purple dot. Periodogram (red lines) was the method with the largest F_1_ score (its optimal point is the one closest to the (1, 1) coordinate). High Freq is plotted in blue. Bottom: Sensitivity and 1-Specificity are employed to build the ROC curves for each method (gray lines). The optimal point of each method according to these metrics (best combination of sensitivity and specificity) is marked with a red dot. Periodogram (red lines) is the method with the highest sensitivity and specificity combination (its optimal point is the one closest to the (0, 1) coordinate). High Freq is plotted in purple. To illustrate that both methods are not equivalent the optimal points for each method employing the PR curves are plotted among the ROC curves (purple) and the optimal points for each method employing the ROC curves are plotted among the PR curves (red).

The optimal parameters belonging to the highest F_1_ score for each method and each iteration are provided in Table 2. After visual inspection of the ROC and PR curves (Fig 4), we noticed a marked difference in the values of precision and recall for most methods. Only High Freq showed a balanced ratio between these metrics. For instance, the optimal point over all PR curves (averaged across iterations) for the classical method with the highest F_1_ score (Welch (2)) had a precision value of 0.92 and a recall value of 0.70 while the optimal point for the parametric method with the highest F_1_ score (High Freq) had a precision value of 0.77 and a recall value of 0.76.

**Table 2:** Optimal parameters for each method.

Method	Lower limit of the tremor bandwidth (Hz)	Power thresholds (m2/s4)
Akaike	2.00–2.40	50
High Amp	2.45–2.90	550
High Freq	2.45–2.90	0
Mode High Amp	2.00–2.40	0–100
Mode High Freq	2.00–2.40	0–100
Periodogram	3.95–4.15	0
Welch (2)	2.95–3.90	0
Welch (3)	3.95–4.35	0
Welch (8)	3.95–4.35	0

Optimal lower limits for the tremor bandwidth and power threshold(s) for each method.

**Fig 5:**
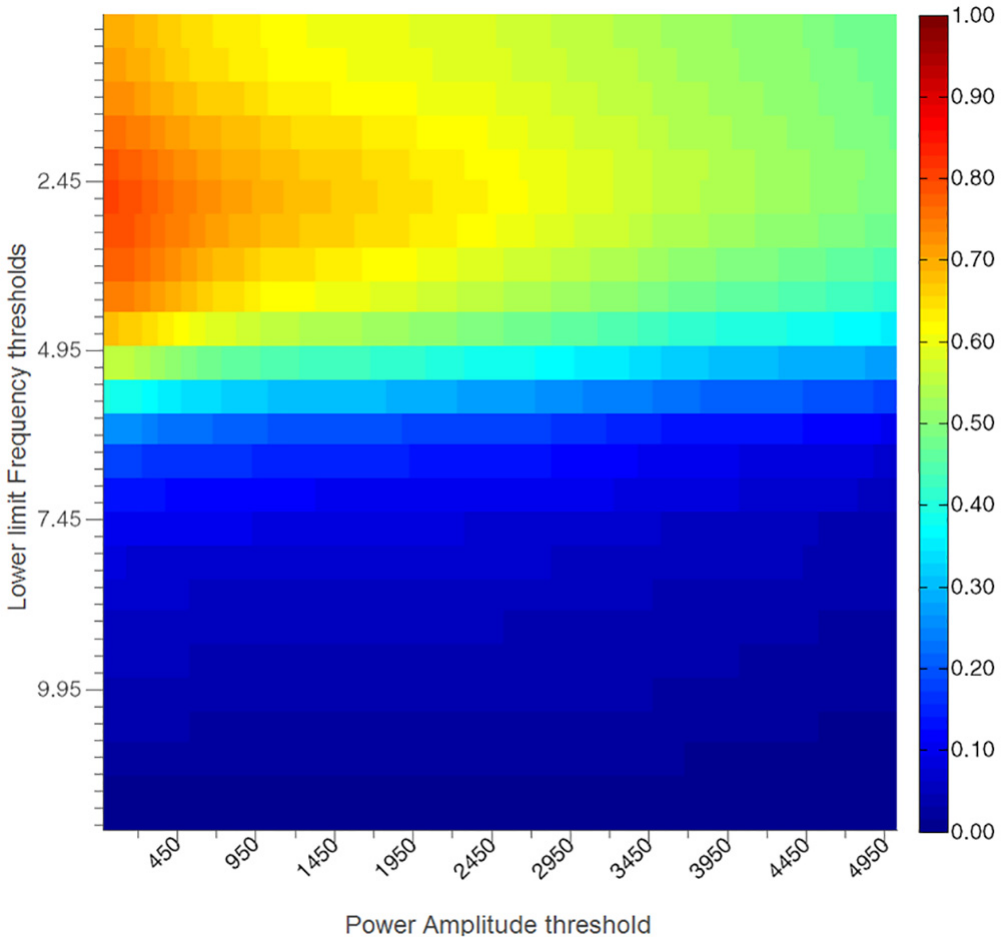
Average F_1_ scores. F_1_ scores for each combination of the lower limit for the tremor bandwidth and power thresholds for the High Freq method averaged across the four iterations. The highest F_1_ score is obtained for lower limits between 2.45–2.90 Hz and a power amplitude threshold of 0 m^2^/s^4^.

The average F_1_ scores for the four iterations and for the possible combinations of lower thresholds and amplitude thresholds of the High Freq method are illustrated in Fig 5.

**Fig 6:**
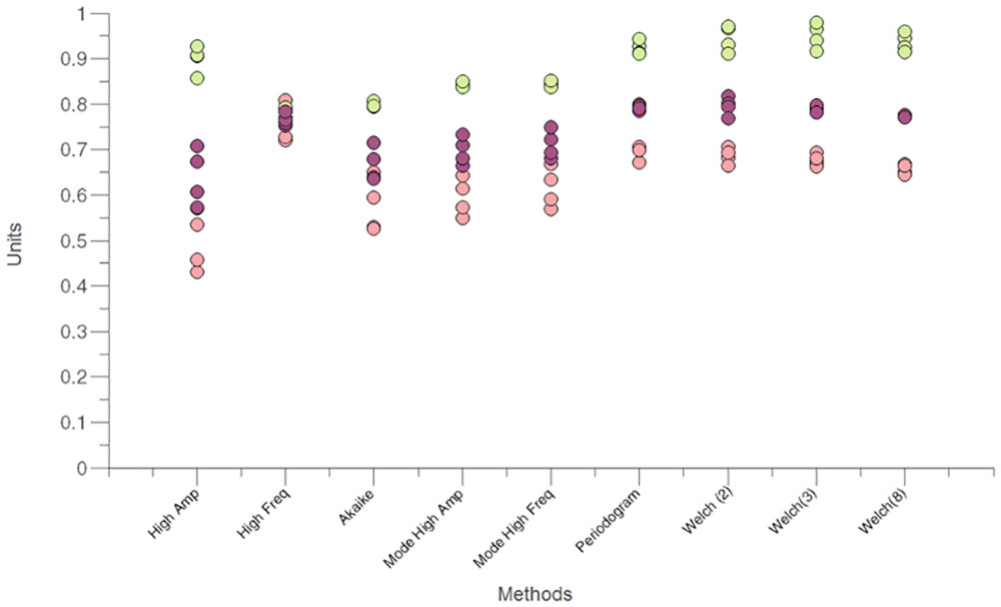
Estimated F_1_ scores. The optimal parameters for each method were derived for each of the four test iterations. The corresponding F_1_ scores (purple), precision (green) and recall (pink) of each fold are plotted. For all methods except High Freq there is considerably higher precision than recall. High Freq is the only method where precision (dots covered by recall dots) and recall have a similar performance.

The results of evaluating each test subset with the optimal parameters of each method are illustrated in Fig 6 and in Table 3. Generally, the method that obtained the highest F_1_ scores on the test subsets was Welch (2) (m = 0.80, std = 0.02). The highest F_1_ score for the parametric methods was obtained for High Freq (m = 0.76, std = 0.01).

**Table 3:** Mean F_1_ score, precision, recall/sensitivity and specificity for each method.

Method	F_1_score	Precision	Recall / Sensitivity	Specificity
Akaike	0.67	0.80	0.57	0.91
High Amp	0.64	0.90	0.50	0.97
High Freq	0.76	0.77	0.76	0.86
Mode High Amp	0.70	0.84	0.60	0.93
Mode High Freq	0.71	0.84	0.62	0.93
Periodogram	0.79	0.92	0.70	0.96
Welch (2)	0.80	0.95	0.69	0.98
Welch (3)	0.79	0.95	0.68	0.98
Welch (8)	0.77	0.94	0.66	0.97

F_1_ score, precision, recall/sensitivity and specificity for each method using their optimal parameters and averaged across patients.

## Discussion

In this study we evaluated the performance of methods for automatically detecting tremor segments using classical and parametric PSD estimation techniques. Our aim was to investigate whether such methods can be used to detect tremor segments in long-term and short-term recordings with similar performance to that of an experienced clinician, using only ACC data.

Overall, non-parametric methods obtained a slightly better performance than parametric methods. This suggests that for this type of analysis, the limited frequency resolution of non-parametric methods due to the four second segment length does not impose any restrictions. As a consequence, and taking into consideration the possibility of suboptimal model selection for parametric methods, we propose employing non-parametric methods for ACC data divided into segments of four seconds or longer.

The High Freq method had a slightly higher average F_1_ score among the parametric methods. This method selects, from the 20 possible models, the model whose peak has the highest frequency. The frequency of this peak is defined as the dominant frequency even if its power is lower than for the other models. It thus tends to select a model that describes a high frequency, which most of the times corresponds to signals within the tremor bandwidth. The generally higher F_1_ score of this method might indicate that clinicians focused their attention on the sometimes subtle presence of tremor to classify a segment as “tremor”.

Most methods displayed a higher precision than recall at the optimal point in the ROC and PR curves. High Freq was the only method that had similar precision and recall at its optimal point (Fig 4). Depending on the type of tremor that is being analyzed, one metric might be more important than the other. For instance, for FT it might be more relevant to identify most of the real tremor segments (high recall) to be able to observe if they are constantly present or intermittent throughout the day. But, if frequency analysis is required, a high precision might be more important, to ensure that the detected tremor segments truly correspond to tremor signals. These performance differences can be derived from the PR curves in Fig 4. It should be noted that precision is influenced by tremor prevalence. The performance obtained in this study may change in a population with a different tremor prevalence. Therefore we can only estimate that a recall (sensitivity) of 0.70 and specificity of 0.96 can be attained using the optimal parameters for the periodogram method in a population with this tremor prevalence. This indicates that 70% of all tremor segments and that 96% of all non-tremor segments present in the signal were correctly classified. For the High Freq method, 76% of all tremor segments and 86% of all non-tremor segments present in our analyzed signals were correctly classified. Method performance was discussed with the neurologists involved in tremor assessment (HvdH and JWE). Due to its relatively high recall and its overall performance, they would prefer High Freq for tremor assessment.

Ideally, our results should be validated in a larger independent patient population. However, in practice it would be hard for movement disorders specialists to evaluate a very large data set using the current evaluation procedure. In the present study, we compromised between the size of the dataset and the time spent on clinical evaluation so that we could provide a first estimate of the performance of different automatic tremor detection methods.

## Conclusion

Generally, non-parametric methods performed better than parametric methods on our data set. Among the parametric methods, High Freq achieved the highest F_1_ score on average. We employed the agreement on the presence or absence of tremor using the visual assessment of two clinicians on ACC data as our reference signal. Our results indicate that, compared to this reference, the automatic detection of a patient’s tremor in this population results in F_1_ scores between 0.64 and 0.80. The proper selection of an automatic tremor detection method depends on the purpose of the study. Even though cross-validation was performed to estimate the performance of each method on new data, validation of our results in longer recordings and with a larger number of patients is still required. After automatic tremor identification, clinicians could focus their analysis on the identified segments and derive other aspects of tremor, like its distribution over time or its frequency variation. This would result in a detailed evaluation of long term recordings of a patient’s tremor without an excessive amount of time spent on analysis by the clinician. This could improve particularly the diagnosis of tremors that have varying characteristics, such as FT.
